# Apical root resorption in Class III patients following pronounced mandibular incisor retraction with lingual high precision fixed appliances: a retrospective cohort study

**DOI:** 10.1186/s13005-025-00577-8

**Published:** 2026-01-08

**Authors:** Julia von Bremen, Dimitrios Kloukos, Collin Jacobs, Lara Bettenhäuser-Hartung, Jonas Q. Schmid

**Affiliations:** 1 Private Practice, Bad Essen, Germany; 2 Department of Orthodontics and Dentofacial Orthopedics, University of Bern, Bern, Switzerland; 3 Department of Orthodontics and Dentofacial Orthopedics, Hellenic Air Force Hospital, Athens, 251 Greece; 4 Department of Orthodontics, Heinrich Heine University, Düsseldorf, Germany; 5 Department of Orthodontics, Hannover Medical School, Hannover, Germany; 6 Department of Orthodontics, University of Münster, Münster, Germany

**Keywords:** High precision fixed appliances, HPFA, Completely customized lingual appliances, CCLA, Orthodontically induced apical root resorptions, OIARR, Class III treatment, Camouflage

## Abstract

**Background:**

Extensive retraction of mandibular incisors in Class III treatment may increase the risk of orthodontically induced apical root resorption (OIARR). This retrospective cohort study aimed to assess the incidence and severity of OIARR in Class III patients treated nonsurgically with lingual high precision fixed appliances (HPFAs) and significant anterior tooth retraction.

**Methods:**

Eligible for inclusion were adolescent and adult Class III patients treated with lingual HPFAs (WIN, DW Lingual Systems GmbH) and extraction of lower premolars, who completed treatment between 2015 and 2024. Pre- (T0) and post-treatment (T1) panoramic radiographs were measured for root and crown lengths, with relative root resorption (rRR, %) calculated for each tooth. Clinically relevant OIARR was assessed using the Malmgren index (scores 1–4). Statistical significance of mean rRR (%) changes was assessed using one-sample t-tests (α = 0.05).

**Results:**

A total of 25 patients (mean age at T1 26.8 ± 9.7 years; 12 females, 13 males; mean Wits at T0 -6.7 ± 2.5 mm) and 350 mandibular teeth were analyzed. The mean rRR for anterior teeth was 3.15 ± 4.05%, with no cases of severe resorption (Malmgren score 4) and only 6.7% of roots exhibiting clinically relevant shortening (Malmgren score 3). There was no increased risk of OIARR in anterior teeth compared to premolars and molars (3.15% vs. 3.31%).

**Conclusion:**

Extensive bodily retraction of lower anterior teeth was not associated with significant OIARR in this Class III cohort. Excellent torque control using HPFAs enabled considerable retraction with low risk of OIARR, supporting this approach as a safe nonsurgical alternative for Class III camouflage.

## Introduction

Orthodontically induced apical root resorption (OIARR) is a well-recognized adverse effect of orthodontic tooth movement and is characterized by the irreversible loss of root structure, predominantly at the apex. Despite improvements in contemporary orthodontic techniques, OIARR remains an unpredictable complication, influenced by a complex interplay of patient-specific biological factors and treatment modalities. In particular, anatomical constraints such as the proximity of tooth roots to the cortical bone have received considerable attention in recent years as a potential contributor to the onset and progression of root resorption [1–4]. The mandibular symphysis forms an important structural boundary that may limit the extent of incisor movement, as authors have found that the lingual cortical bone hinders the movement of lower incisors [5]. When the root apices approach this cortical barrier, the risk of resorptive damage appears to increase as the continued application of orthodontic forces may provoke an excessive compression of the periodontal ligament (PDL), thereby triggering an inflammatory cascade [5].

In 1973, Ackerman and Proffit first introduced the “Envelopes of Discrepancies” to demonstrate how various treatment modalities influence the range of possible orthodontic corrections of different malocclusions [6, 7]. In combination with the use of conventional fixed appliances, they defined three “envelopes”: inner (orthodontics alone), middle (orthodontics + growth modification), and outer (orthodontics + growth modification + orthognathic surgery). These envelopes reflect the capacity for tissue adaptation in relation to increasing treatment invasiveness in combination with conventional fixed appliances. Until now, this model commonly helped clinicians choose among orthodontic, orthopedic and surgical approaches by visualizing the potential and limits of different treatment alternatives. However, with major improvements in the manufacturing of fixed appliances it was suggested that additional potential envelopes that focus exclusively on high precision fixed appliances (HPFA) have to be established [8, 9]. Recently, several studies have underlined the performance of HPFAs, as commonly unexpected tooth movements appear to be possible when high precision third order control is guaranteed, customized bracket prescriptions in combination with completely customized CAD/CAM archwires are used, and the appliances are bonded in a precise indirect bonding process [8–24].

This paper investigates a patient cohort in which the lower incisors were retracted substantially beyond Proffit’s inner envelope to correct a Class III, thereby surpassing the established ‘biological limits’ of tooth movement [8, 9]. Considering that one of the risk factors described for OIARR is root proximity to the cortical bone, it is reasonable to assume that the conducted treatment could bear a high risk for apical root resorption of the lower incisors. Beyond this predisposing factor for OIARR, the cohort also exhibits several additional factors that are well-documented in the literature as contributing to the development of OIARR: (a) major sagittal movements of the apex [25–29], (b) tooth type [4, 29–34], (c) extraction treatment plan [28, 29, 33, 35, 36], (d) Class III malocclusion [31, 35–37], (e) long treatment time [25–27, 37–39]. This study aimed to evaluate the extent of OIARR in a cohort of moderate to severe Class III patients treated with HPFAs, where lower incisors were retracted significantly beyond the original cortical border of the alveolar process after lower premolar extractions. Given the numerous contributing factors within this cohort, it can be classified as a high-risk sample for OIARR. The null hypothesis was that there is no significant change in root length in Class III patients treated with HPFAs and pronounced incisor retraction.

## Materials and methods

The approval for this retrospective cohort study was received from the ethical committee of the Hannover Medical School, Hannover, Germany (3151–2016). This study is a follow-up evaluation of the studies of Thiem et al. and Wiechmann et al. and included the patient sample described in detail there [8, 9]. Briefly summarized, adolescent or adult Class III patients treated consecutively with high precision fixed appliances (HPFA; WIN, DW Lingual Systems GmbH, Bad Essen, Germany), and lower bicuspid extraction in one orthodontic specialist practice (Bad Essen, Germany), whose treatment was completed between 2015 and 2024 were eligible for inclusion.

Baseline characteristics that could be prognostic for the outcome were controlled with strict inclusion criteria:

- Class III molar relationship (≥ ½ cusp) on one or both sides.
- Wits value of ≤ −2 mm.
- uni- or bilateral lower first or second premolar extraction without counterbalancing extractions in the maxilla.
- mandibular third molars present without displacement or visible pathology.

**Fig. 1:**
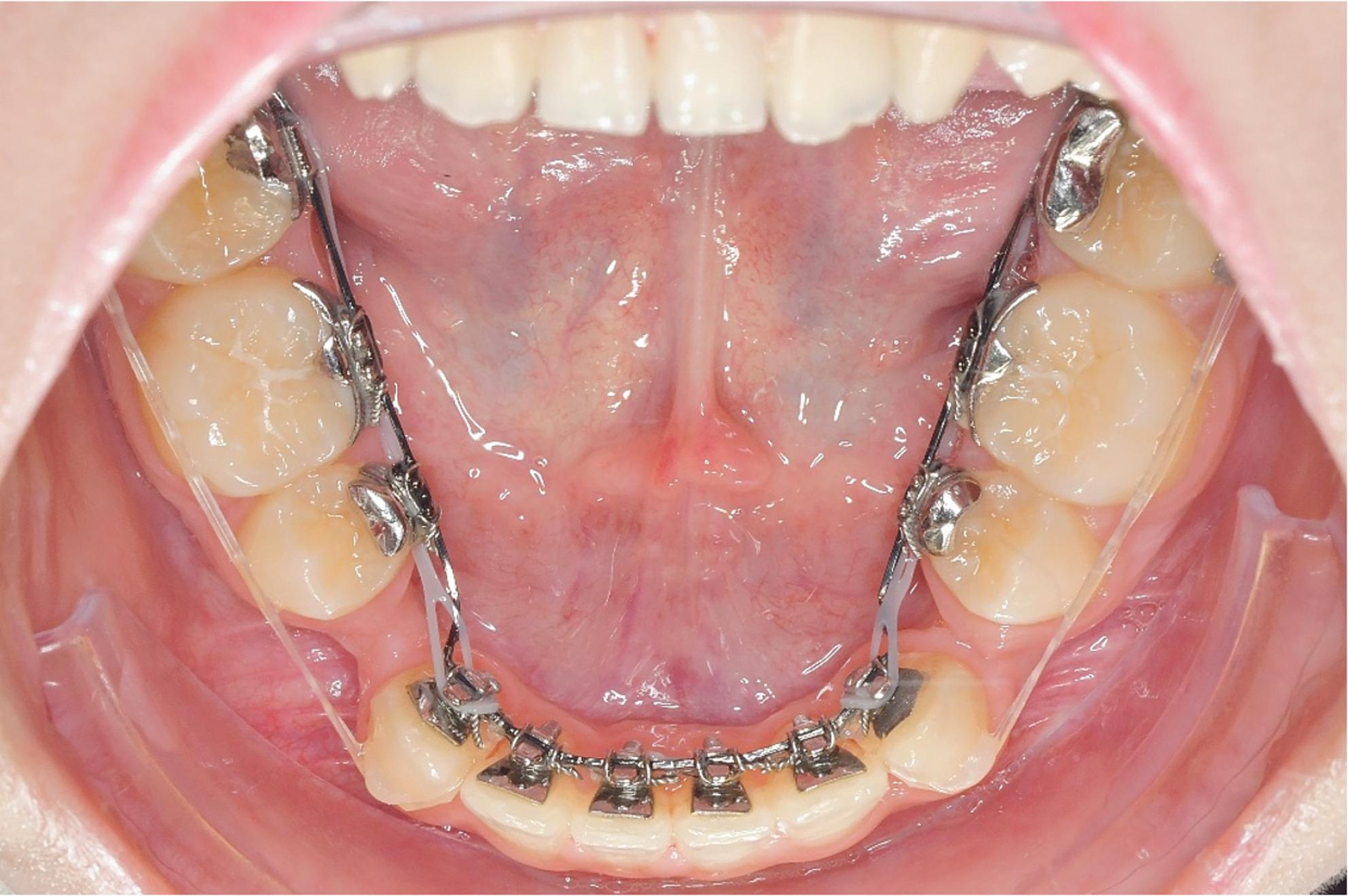
Double cable approach for mandibular space closure

No patient was excluded from the consecutive sample for any reason (e.g. bad compliance, missing records, bad oral hygiene or missing appointments), however, 5 teeth with an incomplete root development at the start of treatment were excluded from further analyses. The treatment plan with lower premolar extractions on one or both sides was defined by an individual target set-up. En masse space closure in the mandible was performed using 0.016’’ × 0.024’’ ribbonwise stainless steel archwires with 13° of positive crown torque in the anterior segment from canine to canine. If necessary, this extra-torque was upscaled to 21°. Power chains were used for space closure, mostly in a double cable approach (Fig. 1). Intermaxillary Class II or Class III elastics were prescribed if necessary to modulate anchorage.

**Fig. 2:**
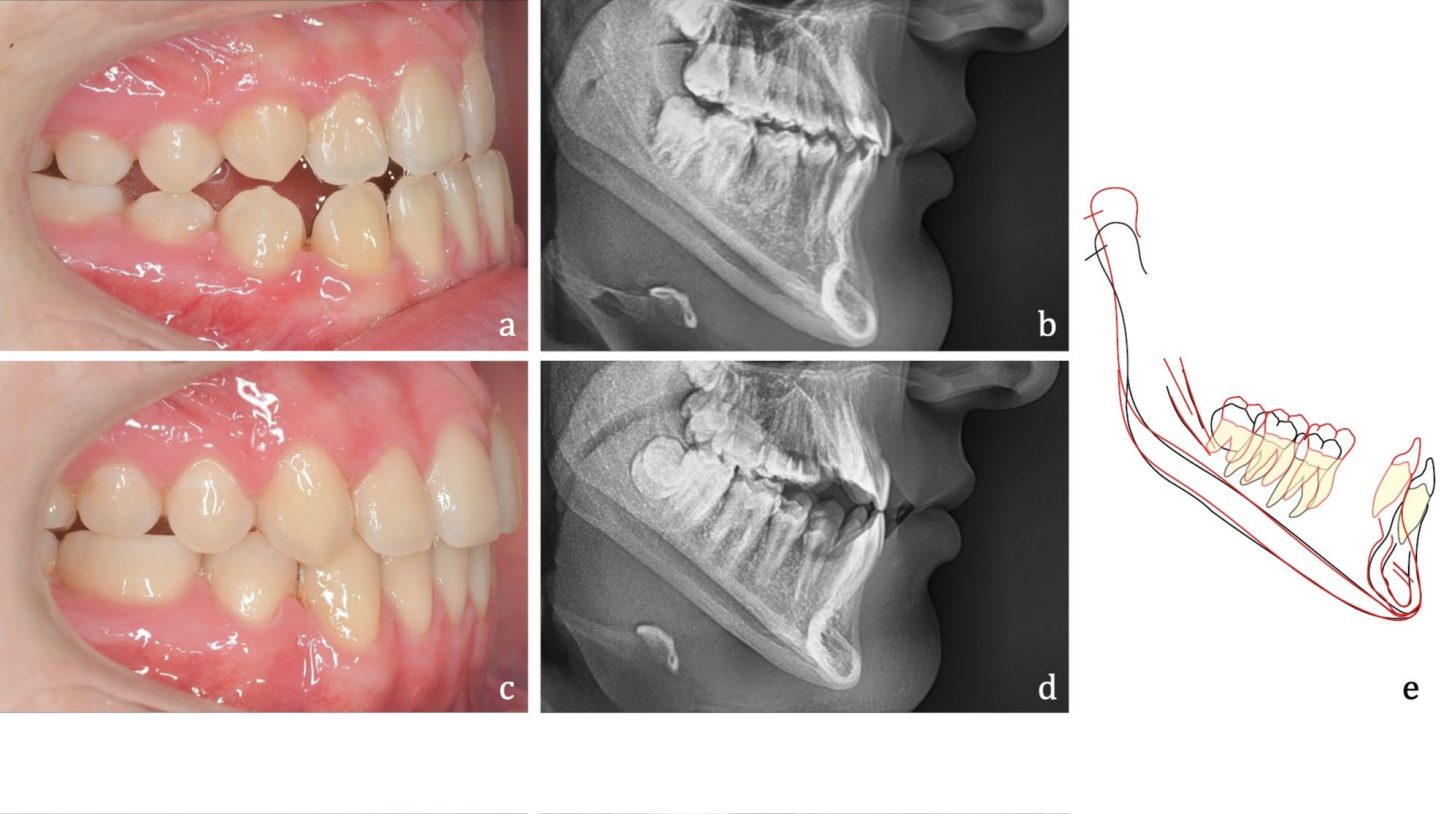
Adult study participant with Class III malocclusion. **a**, **b** Situation before camouflage treatment treatment. **c**, **d** Situation after treatment with HPFAs. **e** Stuctural superimposition of the mandible to visualise the treatment effects

A typical example of a patient of the present cohort before (T0) and after (T1) treatment is shown in Fig. 2.

### OIARR assessment

**Fig. 3:**
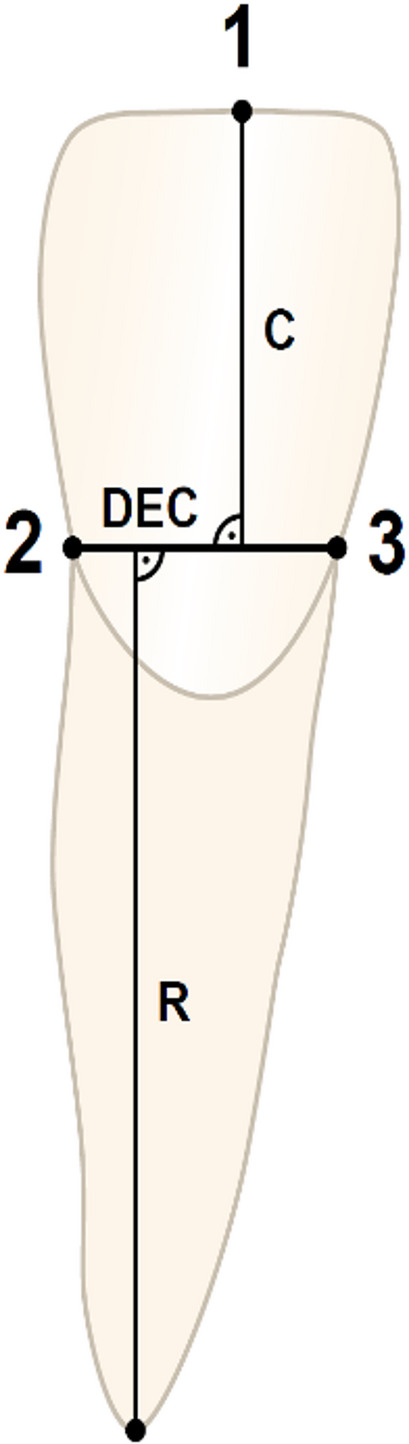
OIARR Measurements according to Linge and Linge [40]. Reference points: 1 incisal edge, 2 distal dento-enamel junction, 3 mesial dento-enamel junction, 4 root apex. Dento-enamel conjunction (DEC) represents the conjunction between mesial and distal. Crown length (C) and root length (R) were measured perpendicular to DEC as the longest distance to the root apex and the incisal edge

The pre- (T0) and post-treatment (T1) panoramic radiographs were used to assess possible OIARR on the teeth according to the method first described by Linge and Linge [40] and since then applied in several studies [35, 40–42] (Fig. 3). Quantitative measurements of the crown and root length of the mandibular incisors and canines were performed, taking possible image distortion between the pre- and post-treatment radiographs into account using crown length registrations according to the following calculation:

$$\mathrm{Correction}\;\mathrm{Factor}\;(\mathrm{CF})\;=\;\mathrm C1/\mathrm C2$$

$$\mathrm C1=\mathrm{Crown}\;\mathrm{length}\;\mathrm{on}\;\mathrm{pretreatment}\;\mathrm{radiograph}$$

$$\mathrm C2=\mathrm{Crown}\;\mathrm{length}\;\mathrm{on}\;\mathrm{post}-\mathrm{treatment}\;\mathrm{radiograph}$$

The OIARR in millimeters was calculated as follows:

$$\mathrm{OIARR}=\mathrm R1-\;(\mathrm R2\;\mathrm x\;\mathrm{CF})$$

$$\mathrm R1=\mathrm{Root}\;\mathrm{length}\;\mathrm{before}\;\mathrm{treatment}$$

$$\mathrm R2=\mathrm{Root}\;\mathrm{length}\;\mathrm{after}\;\mathrm{treatment}$$

The OIARR was described as the relative root resorption (rRR) seen as the percentage shortening per tooth:

$$\mathrm{rRR}\;(\mathrm{Resorption}\;\mathrm{per}\;\mathrm{tooth}\;\mathrm{in}\;\%)\;=\;(\mathrm{OIARR}\;\mathrm x\;100)/\mathrm R1$$

**Fig. 4:**
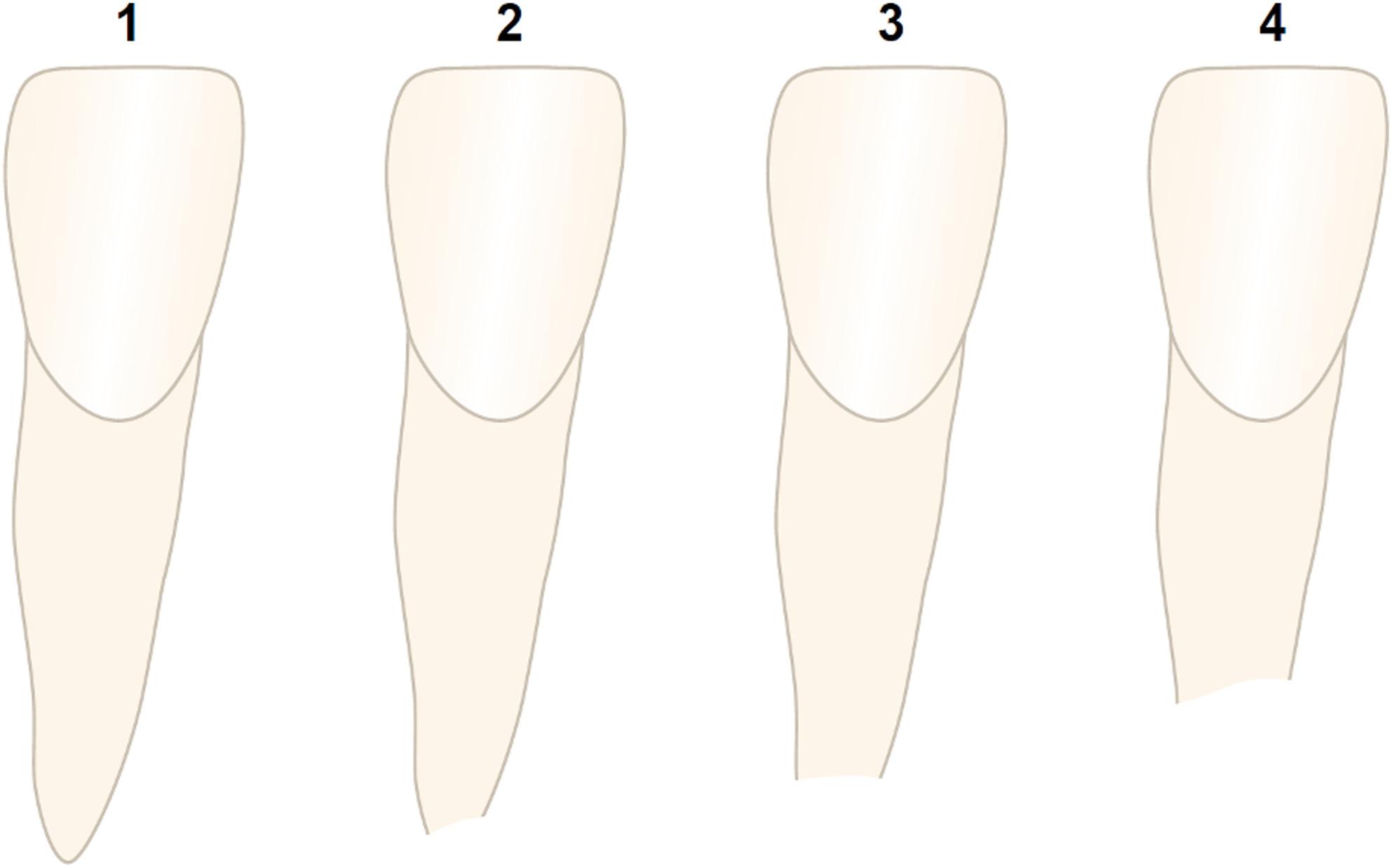
External apical root resorption (EARR) stages based on Malmgren et al. [43]. 1: irregular root contour; 2: EARR < 2 mm of root length; 3: EARR > 2 mm to 1/3 of root length; 4: EARR > 1/3 of root length

In addition to these measurements, the Malmgren index was applied to detect clinically relevant root resorptions [43, 44]. This is a four-score index (1–4) categorizing root resorption from none to severe (Fig. 4), which is commonly applied in international literature [44–47]. Whereas grades 1 and 2 describe an irregular root contour or an apical resorption up to 2 mm, respectively, grade 3 describes a clinically relevant resorption of up to 1/3 root length. According to Malmgren et al., a severe OIARR is defined as grade 4 root resorption, characterized by resorption of more than one-third of the root length (i.e., rRR > 33.33%). To ensure validity of the measurements and inter-observer reliability, two experienced orthodontists performed all measurements independently from one another (J.B., C.J.). Intra-rater reliability was assessed by randomly selecting 10% of the sample for re-evaluation by rater 1 at least 4 weeks after the initial measurements.

### Statistical analysis

Inter-rater reliability was evaluated by independent assessment of all patients by both raters (rater 1 and rater 2). For rRR (%) measurements, intraclass correlation coefficients (ICC), specifically ICC (3,1) as described by Shrout and Fleiss [48], were calculated. Agreement in Malmgren scores between raters was assessed using weighted Cohen’s kappa with 95% confidence intervals, interpreted according to Landis and Koch [49].

Intra-rater reliability for the percentage change in root length (rRR (%)) was estimated using ICC (3,1) as above, with interpretation of ICC values according to the cut-off criteria described by Koo and Li (2016) [50]. Measurement error for intra-rater assessments was evaluated using the method described by Dahlberg [51].

Descriptive statistics were calculated for rRR (%) using mean, ± standard deviation (SD), median, minimum (min), and maximum (max). Categorical data (Malmgren scores) were reported as frequencies and percentages. Analyses were performed on measurements made by rater 1. Results from rater 2 were used exclusively to assess inter-rater reliability.

The primary endpoint, the percentage change in root length (rRR (%)), was analyzed using one-sample t-tests to test the null hypothesis that the mean rRR (%) was equal to zero (H0 = 0), with a significance level of α = 0.05.

A *p*-value *p* < 0.05 was considered as statistically significant. Based on the explorative nature of this study, no alpha correction was applied. All statistical analysis were conducted using SAS version 9.4 (SAS Institute, Cary, NC, USA).

## Results

The baseline characteristics (T0) are summarized in Table 1.

**Table 1:** Baseline characteristics

Included patients (*n*)	25
Evaluated teeth (*n*)	350
Evaluated roots (*n*)	450
Gender *n* (%)	
Female	12 (48%)
Male	13 (52%)
Wits females at T0 (mm)	
Mean ± SD	−6.3 ± 2.5
Min/Max	−10.8/−3.4
Wits males at T0 (mm)	
Mean ± SD	−7.1 ± 2.5
Min/Max	−10.7/−2.1
Age at T1 (years)	
Mean ± SD	26.8 ± 9.7
Min/Max	16.3/49.5

The inter-rater reliability for the Malmgren scores, as assessed by the weighted Cohen’s Kappa coefficient, was 0.81 (95% CI [0.75, 0.87]), indicating almost perfect agreement. The intraclass correlation coefficient (ICC) for inter-rater reliability was 0.84 for relative root resorption percentage (rRR (%)), which indicates good reliability. Consequently, only the measurements from one rater (JB) were used for further analyses. The intra-rater reliability for rater JB was also good, with an ICC of 0.78 for rRR (%)). The method error for intra-rater assessments, calculated by Dahlberg’s formula, was 3.63% for rRR (%).

**Fig. 5:**
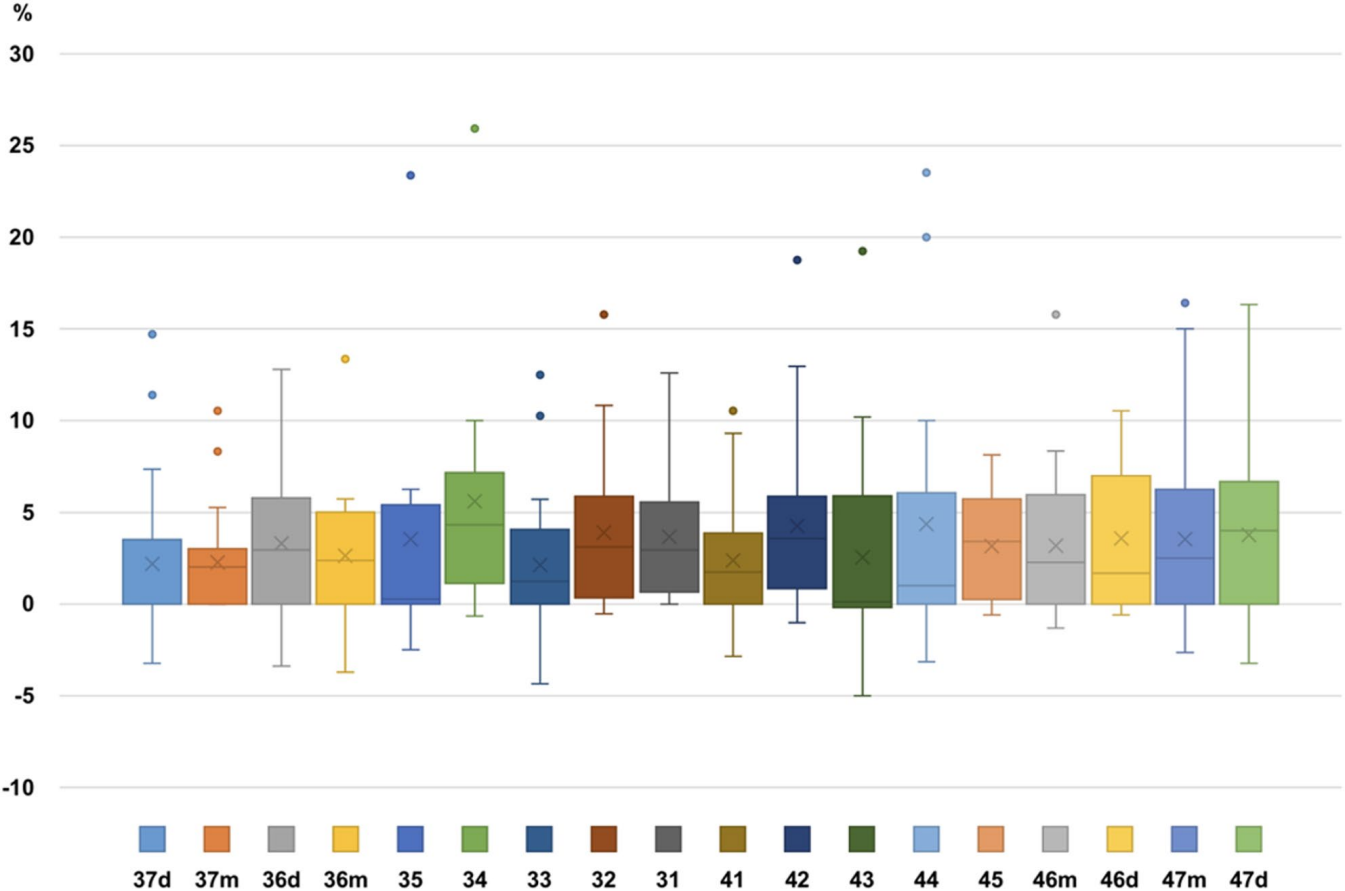
Distribution of mean rRR (%) per root

The mean relative change of root length (pre-post treatment) for the different teeth of all patients was between 2.1% and 5.6% with a mean of 3.3% and very high inter-individual variation, as can be assessed in the box plots in Fig. 5. No tooth group (incisors, canines, premolars, molars) seemed to be at a particular risk for OIARR (Fig. 5).

**Fig. 6:**
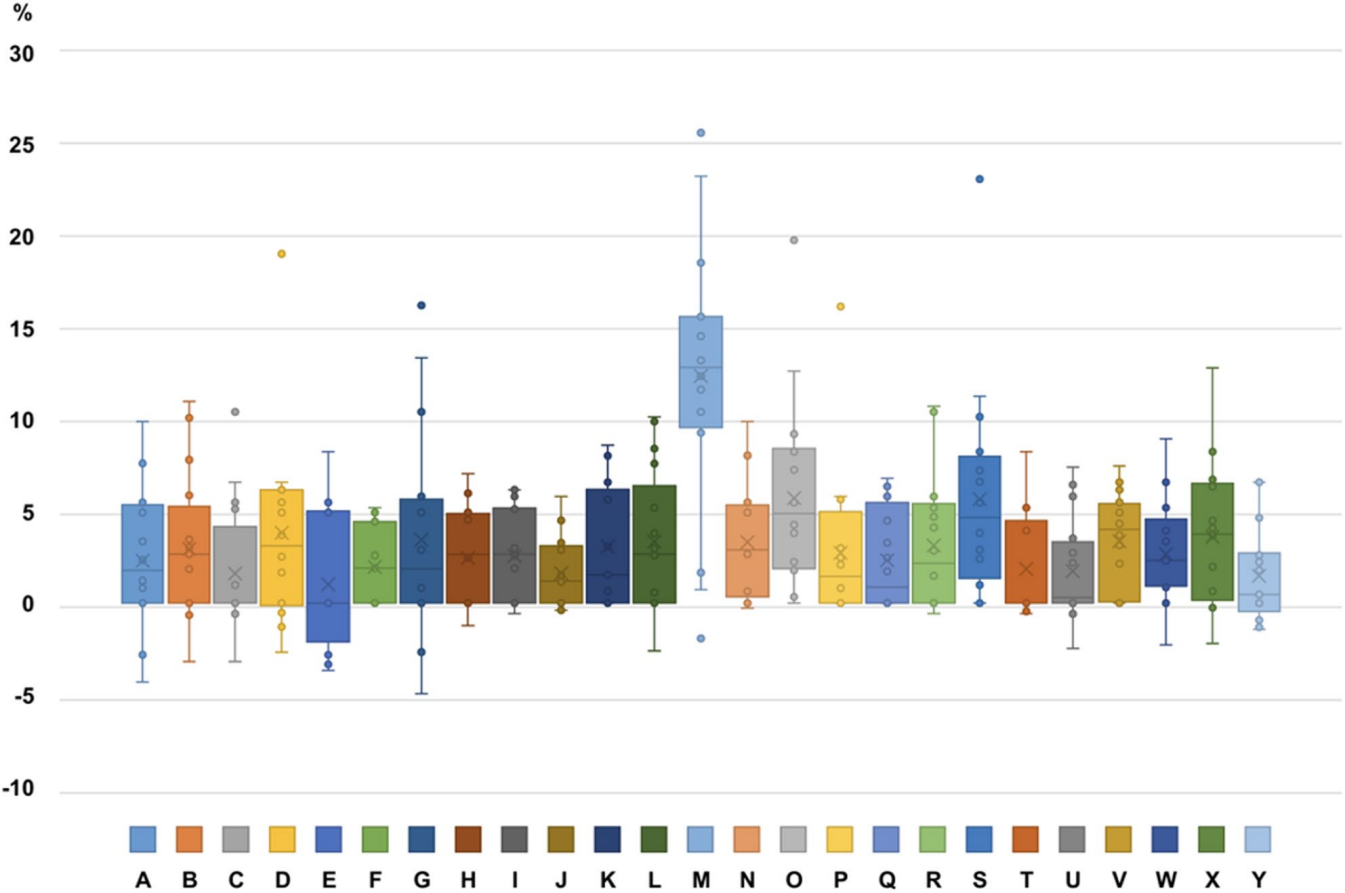
Distribution of mean rRR (%) per patient

Whereas the majority of patients (22/25, 88%) had a mean rRR of less than 4%, one patient (4%) had a mean rRR of 12.5% and two others of 5.8% and 5.7%, respectively (Table 2; Fig. 6). The relatively high value of > 10% root shortening in patient “M” reflects the noticeable generalized resorptions in this patient, with 8/12 teeth presenting with a Malmgren Score of 3 at T1.

**Table 2:** Descriptive table showing mean root length change (%) per patient

Patient	*N*	Mean	SD	Median	Min	Max
A	16	2.34	3.76	1.81	−4.35	10
B	16	2.99	4	2.7	−3.23	11.11
C	16	1.63	3.51	0	−3.23	10.53
D	14	3.88	5.37	3.16	−2.7	19.25
E	16	1.03	3.73	0	−3.72	8.33
F	15	1.99	2.17	1.94	0	5.26
G	16	3.49	5.81	1.89	−5	16.41
H	16	2.49	2.7	2.68	−1.25	7.14
I	16	2.6	2.53	2.71	−0.59	6.25
J	16	1.65	1.94	1.21	−0.39	5.88
K	13	3.11	3.42	1.56	0	8.7
L	17	3.39	3.8	2.7	−2.63	10.26
M	16	12.51	7.54	12.99	−1.95	25.93
N	17	3.36	3.17	2.94	−0.27	10
O	16	5.79	5.29	4.94	0	20
P	16	2.77	4.24	1.47	0	16.34
Q	16	2.36	2.79	0.88	0	6.88
R	16	3.18	3.75	2.2	−0.59	10.84
S	16	5.73	5.91	4.71	0	23.37
T	14	1.88	2.91	0	−0.59	8.33
U	16	1.76	2.86	0.31	−2.5	7.5
V	16	3.41	2.67	4.06	0	7.57
W	17	2.7	2.81	2.34	−2.31	9.04
X	16	3.67	3.93	3.82	−2.22	12.96
Y	17	1.5	2.59	0.48	−1.45	6.67

Concerning the patient‑level prevalence of Malmgren ≥ 3 it was found that 11 patients (44%) did not have one single tooth classified as grade 3, 10 patients (40%) had one tooth classified as grade 3, three patients (12%) 2 teeth and one patient (4%) had 7 teeth categorized as Malmgren Score 3 (Table 3).

**Table 3:** Patient-level prevalence of Malmgren score 3 concerning the number of affected teeth

Number of teeth classified as Malmgren_score 3	Patients (*n*)	Percent (%)
0	11	44
1	10	40
2	3	12
7	1	4

### Anterior teeth (33-43)

A special focus was put on the roots of the lower incisors and canines, as these seem to have undergone the most critical tooth movement, with a bodily retraction of several millimeters. Here, the mean root resorption was 3.2 ± 4.05% compared to an average root resorption of 3.3 ± 4.64% for the premolars and molars, indicating no higher risk for OIARR for the anterior teeth, but a statistically significant apical root resorption relative to T0 (Table 4).

**Table 4:** Average root length change (%) for all evaluated roots (pre-post treatment) analyzing anterior teeth vs. posterior teeth, as well as paired t-Test for the H0: difference = 0

Variable/Tooth	*N*	Mean	SD	95% CI	Minimum	Maximum	*p*-Value
rRR (%) 33–43	149	3.15	4.05	(2.49, 3.80)	−5.00	19.25	< 0.001
rRR (%) 37 − 35, 45–47	247	3.31	4.64	(2.73, 3.90)	−3.72	25.93	< 0.001
rRR (%) Total	396	3.25	4.42	(2.81, 3.69)	−5.00	25.90	< 0.001

### Malmgren score

Although statistically significant, a clinically relevant root resorption (Malmgren Score 3) was only found for 6.7% of the lower incisors and canines compared to 93.3% of the anterior teeth with a score of ≤ 2. Not a single tooth presented a resorption categorized as score 4 (Tables 5 and 6).

**Table 5:** Grouped Malmgren scores for the 6 anterior teeth (33–43)

Malmgren score	Frequency	Percentage	Cumulative Frequency	Cumulative Percentage
≤ 2	139	93.29	139	93.29
≥ 3	10	6.71	149	100.00

**Table 6:** Malmgren score distribution for all teeth

Tooth/Variable	Malmgren Score				
Frequency Row Percentage	1	2	3	4	Total
31 Malmgren	15 60.00	8 32.00	2 8.00	0 0.0	25
32 Malmgren	14 56.00	9 36.00	2 8.00	0 0.0	25
33 Malmgren	17 68.00	7 28.00	1 4.00	0 0.0	25
34 Malmgren	7 50.00	5 35.71	2 14.29	0 0.0	14
35 Malmgren	9 64.29	4 28.57	1 7.14	0 0.0	14
36 Malmgren	15 60.00	8 32.00	2 8.00	0 0.0	25
37 Malmgren	18 75.00	5 20.83	1 4.17	0 0.0	24
41 Malmgren	17 68.00	8 32.00	0 0.00	0 0.0	25
42 Malmgren	11 44.00	11 44.00	3 12.00	0 0.0	25
43 Malmgren	16 64.00	7 28.00	2 8.00	0 0.0	25
44 Malmgren	9 64.29	3 21.43	2 14.29	0 0.0	14
45 Malmgren	5 38.46	8 61.54	0 0.00	0 0.0	13
46 Malmgren	13 52.00	9 36.00	3 12.00	0 0.0	25
47 Malmgren	13 52.00	10 40.00	2 8.00	0 0.0	25
Total	179	102	23	0	304

## Discussion

It is notable that in the present patient cohort, despite mandibular incisor retractions often exceeding 5 mm in the center of resistance, the mean OIARR was under 5%, with fewer than 7% of roots showing clinically significant root shortening (> 2 mm, Malmgren score 3) and none have > 1/3 root length resorption (Malmgren score 4) on any tooth.

Previous studies have found that roots located within 1 mm of the labial or lingual cortical plates are at elevated risk for OIARR [3]. In the present sample, however, only minimal root resorption was observed, despite a short apex-to-cortex distances or even direct cortical contact. This suggests that proximity alone may not be a determinative factor; rather, the quality and remodeling capacity of the cortical and cancellous bone may play key roles. In their study on cone beam CTs (CBCT), Wang et al. also performed a significant lingual movement of the lower incisor crowns (tipping movement) and observed a strong bone remodeling ability in the upper part of the alveolar process, even if roots had penetrated the upper part of the original lingual border [52]. Despite roots being moved outside the cortical bone, new lingual bone apposition occurred in the majority of their patients.

Due to radiation protection and in accordance with the “as low as reasonably achievable” (ALARA) principle, the indication for performing CBCTs in the current setting (Germany) is very strict, so these images are not taken on a routine basis. Naturally, it would have been interesting to conduct a detailed 3D analysis of root morphology and bone supply, but this was not possible due to ethical reasons.

It can be discussed that the complete torque control achieved through the HPFA (and with this the bodily movement of the teeth) might be associated with a reduced root resorption compared to tipping movements, due to a more balanced force distribution. By such, not a single root is moved into the cortical bone, but rather the whole block of anterior teeth seems to relocate the alveolar process posteriorly. Animal studies have shown that during tooth movement, it is crucial to apply light and continuous forces to preserve the marginal bone and to prevent unwanted resorption [53], especially when movement occurs against the cortical bone [54]. If the tooth moves parallel within the bone, without excessive tipping, the force is more evenly distributed across the bone and periodontal ligament. This contributes to generalized lighter forces and more predictable bone remodeling, which reduces the risk of undesirable side effects, such as marginal bone loss and root resorption [55]. Such movements demand precise torque control—this is exactly where the challenge lies, as the slot dimension of conventional bracket systems is generally imprecise and therefore insufficient to enable complete en-bloc retraction without undesired incisor tipping [56].

As seen in the superimpositions of the analyzed patients the entire symphysis is relocated posteriorly in many cases [9]. Although this appears extreme in our usual everyday practice, it has to be remarked that bone remodeling and morphological adaptations beyond the limits of the bony envelope as a consequence to environmental and functional changes are not unusual in general. Such observations are particularly evident in patients with neuromuscular disorders, where altered muscle function and strength significantly impact jawbone morphology [57, 58]. This underlines the high ability of the bone to remodel according to functional requirements.

Whereas in general, the fixed treatment performed in the present patient cohort did not lead to significant root resorptions, it should be reported that one single patient exhibited generalized OIARR after treatment (Fig. 4). This once more shows that the individual biological response to orthodontic forces, mediated by a network of cytokines and osteoprotegerin, plays a crucial role in the resorptive process and is not always predictable. A certain predisposition may always interact with biomechanical factors, resulting in substantial inter-individual variability regarding the severity of resorption. Several systematic reviews conclude that the etiology of OIIRR is multifactorial: force magnitude and duration, individual susceptibility, and anatomical constraints all interplay [28, 59–61]. As it seems clinically impossible to assess all potential risk factors prior to orthodontic treatment, a certain risk of OIARR naturally always remains. Generally, it has to be pointed out, however, that not one single patient in the present sample presented a severe OIARR according to Malmgren (> 1/3 root length resorbed). Considering the potential risks of orthognathic surgery, which would have been the only treatment alternative for the present patient cohort, extraction treatment and alveolar process remodelling with HPFAs offers a viable option for class III patients whose facial profile is not their main concern, as the amount of root resorptions is no higher than in any other orthodontic therapy with multibracket appliances.

### The concept of High Precision Fixed Appliances (HPFA)

For the present patient cohort, the findings of Thiem et al. [8], describing a highly unusual average uprighting of the mandibular incisors and those of Wiechmann et al. [9] documenting a substantial displacement of the entire mandibular alveolar processes both describe results not previously reported in the literature. In combination with the results of the present study, indicating that these remarkable tooth movements were possible without a particularly high OIARR risk, the question which arises is if these movements would have been possible with conventional fixed appliances. Several authors have emphasized that the use of HPFAs allows for a precise transfer of the planned target setup occlusion into the patient’s mouth [10–12]. This level of precision, combined with appliance customization, makes it possible to perform previously uncommon tooth movements as a reliable and predictable option in contemporary treatment planning. As conventional fixed appliances generally work with average torque- and angulation values plus oversized bracket slots, it is difficult to achieve completely controlled tooth movements towards an individually planned target occlusion using this approach. Thus, it seems more predictable and more controlled to perform such large tooth movements with HPFAs. Table 7 summarizes the major differences between conventional fixed appliances and HPFAs.

**Table 7:** Differences between conventional fixed appliances and HPFAs

	Conventional Fixed Appliances	High Precision Fixed Appliances
Target occlusion/Prescription	Average values for torque in/out and angulation	Values of an individual target set-up
Slot precision	5–24% oversized (mostly divergent)	Average oversize < 0.1%, parallel walls
Target archform/Archwire shape	Prefabricated standard archwires	Archwire shape based on individual target set-up
Bracket placement errors	Possible through direct bonding	Unlikely, due to indirect bonding with full arch transfer tray
Treatment philosophy	Use undersized archwires	Use full size wires or undersized wires with extra-torque to avoid toque play

### Strengths and limitations

Generally, it has to be remarked that panoramic radiographs are less precise than periapical radiographs or CBCTs for the determination of root resorptions, but due to the retrospective study set-up, these data did not exist [62]. As the present subject material consisted of standard diagnostic records, panoramic radiographs were available and considered adequate and sufficient for answering the present research question. As the main disadvantage of them is a distortion due to the magnification or inclination change, it was refrained from evaluating metrically, but rather, like in many other investigations, chosen to use relative values and classify the findings into resorption stages [35, 40–44]. By measuring the crown/root-ratio a presumably shortened root (e.g. caused by a proclination of incisors) does not distort the relative values.

The measurement error (Dahlberg error of 3.63% for rRR) was small in relation to the amount of root resorption observed in this study, indicating that our measurements were sufficiently accurate to detect the reported effects. However, as teeth are clustered within patients, our analysis does not account for potential intra-patient correlation, which may have led to underestimation of variability and *p*-values. We also did not report the median or interquartile range, which may limit interpretation of the effect size and distribution.

In summary, although proximity to the cortical bone of the mandibular symphysis is a recognized risk factor for orthodontically induced root resorption, our findings demonstrate that controlled biomechanics and the remodeling capacity of the cortical and cancellous bone can preserve root integrity even during substantial incisor bodily retraction. These results indicate that the range of tooth movement can be expanded through en-masse retraction under complete torque control with HPFAs, thereby redefining the limits of orthodontic tooth movement. However, future studies should include a control group treated with conventional fixed appliances to directly compare the risk and extent of root resorption between appliance systems and more robustly assess the potential benefits of high precision fixed appliances.

## Conclusion

Extensive bodily retraction of lower anterior teeth was not associated with significant OIARR in this Class III cohort. Excellent torque control using HPFAs enabled considerable retraction with low risk of OIARR, supporting this approach as a safe nonsurgical alternative for Class III camouflage.

## Data Availability

The datasets used and/or analysed during the current study are available from the corresponding author on reasonable request.
